# A New Microwave Shield Preparation for Super High Frequency Range: Occupational Approach to Radiation Protection 

**Published:** 2016-11-22

**Authors:** Vida Zaroushani, Ali Khavanin, Ahmad Jonidi Jafari, Seyed Bagher Mortazavi

**Affiliations:** ^a^ Department of Occupational Health Engineering, School of Public Health, Qazvin University of Medical Sciences, Qazvin, Iran; ^b^ Department of Occupational Health Engineering, School of Medical Sciences, Tarbiat Modares University, Tehran, Iran; ^c^ Research Center for Environmental Technology, Iran University of Medical Sciences, Tehran, Iran; ^d^ Department of Environmental Health Engineering ,School of Public Health, Iran University of Medical Sciences, Tehran, Iran; ^e^ Department of Occupational Health Engineering, School of Medical Sciences, Tarbiat Modares University, Tehran, Iran

**Keywords:** Epoxy Resin, Radiation Protection, Nickel Oxide, Microwave

## Abstract

**Background:** Widespread use of X-band frequency (a part of the super high frequency microwave) in the various workplaces would contribute to occupational exposure with potential of adverse health effects. According to limited study on microwave shielding for the workplace, this study tried to prepare a new microwave shielding for this purpose.

**Methods:** We used EI-403 epoxy thermosetting resin as a matrix and nickel oxide nanoparticle with the diameter of 15-35 nm as filler. The Epoxy/ Nickel oxide composites with 5, 7, 9 and 11 wt% were made in three different thicknesses (2, 4 and 6 mm). According to transmission / reflection method, shielding effectiveness (SE) in the X-band frequency range (8-12.5 GHz) was measured by scattering parameters directly given by the 2-port Vector Network Analyzer. The fabricated composites characterized by X-ray Diffraction and Field Emission Scanning Electron Microscope.

**Results:** The best average of shielding effectiveness in each thickness of fabricated composites obtained by 11%-2 mm, 7%-4 mm and 7%-6 mm composites with SE values of 46.80%, 66.72% and 64.52%, respectively. In addition, the 11%-6 mm, 5%-6 mm and 11%-4 mm-fabricated composites were able to attenuate extremely the incident microwave energy at 8.01, 8.51 and 8.53 GHz by SE of 84.14%, 83.57 and 81.30%, respectively.

**Conclusions:** The 7%-4mm composite could be introduced as a suitable alternative microwave shield in radiation protection topics in order to its proper SE and other preferable properties such as low cost and weight, resistance to corrosion etc. It is necessary to develop and investigate the efficacy of the fabricated composites in the fields by future studies.

## Introduction

Microwave radiation is a kind of electromagnetic radiation in the frequency range from 300 MHz to 3000 GHz. X-band frequency (8-12.5 GHz) is part of super high frequency microwave radiations with various applications such as satellite communications, radar, navigation, air traffic control, marine, military and weather station ^1^. Therefore, many workers are exposed to X-band frequency at the workplace ^2^.

The electromagnetic radiation is one of the adverse physical agents in the workplace^3. ^There are many studies on exposure assessment and biological effects of microwave radiation^4^. However, there are few studies about radiofrequency (RF) and microwave radiation shielding carried out in lower frequency (megahertz) not in super high frequency microwave radiations^5^.

Compliance of recommended exposure limits is the most administrative control reported so far. This limited exposure time is a restriction in the workplace that may not always be possible. Therefore, investigation of microwave shielding is necessary and best approach to non-ionizing radiation protection.

Previously, metal panel/sheet^1^, wire mesh/ metal screen/ fence^6^, metal foil^7^ usually have been used to provide favorable shielding against RF/microwave radiation but for lower frequencies^8^. Shields made of metal have very limited applicable because their properties: costly, high weight, not easy usable and low resistance to corrosion^9^. Therefore, it is necessary to develop the microwave shielding in the workplace by alternative material or a shield to dissolve disadvantage of mentioned metallic shields.

A good shielding material should restrict both entering and outgoing the electromagnetic radiation. There are three main mechanisms for electromagnetic shielding that including: Absorption (A), Reflection (R) and Multiple Reflection (B)^10^. Absorption results from electric or magnetic dipoles in the shield material. Reflection is another mechanism of electromagnetic shielding that results from an impedance mismatch between wave impedance of free space and essential impedance of the shield^11^.

With progression in nanotechnology, the composites made of polymer-matrix have important role in developing new microwave shielding materials. Nowadays, microwave shields include the various polymer matrix such as epoxies, elastomers or silicones because of their lightweight, resistance to corrosion, ﬂexibility and easy to production^12^.

In mentioned polymer, Epoxy resins - a thermosetting polymer- duo to some favorable properties such as good dielectric constant and excellent electrical properties lead to carried out the very wide research about electromagnetic shielding^13^. Besides, our previously study showed that epoxy resin could be a shielding material for microwave with reflection as the first and main mechanism and absorption as the secondary mechanism of electromagnetic shielding^14.^

In recent years, different types of metal based nanoparticles such as ferrites, metals, metal oxides and metal alloys have been extensively used as filler^11^. Nickel oxide (NiO) is a metal oxide with paramagnetic and p-type semiconductor properties used in optical, electronic, catalytic and super-paramagnetic devices ^15^. However, electromagnetic shielding approach with the use of NiO in bulk or nano scales had not been previously investigated. According limited studies about shielding design / or a provision for microwave and a significant lack of shielding in higher frequencies for the workplace, this study tried to prepare a new electromagnetic shield (using epoxy resin / nano nickel oxide) to reduce occupational exposure of X-band frequency range in the workplace.

## Methods

### Materials preparation

We used *”EI-403”* a two component epoxy thermosetting resin (Mokarrar Manufacturing Co. Iran) with characters indicated earlier ^16^ as a matrix and nickel oxide nanoparticle with the diameter of 15-35 nm as a filler. According to the supplier product information (U.S Research Nanomaterial Co. USA), all particles have a near spherical shape and a purity of at least 99.5%. The manufacturing method applied for particle synthesis was "Laser Evaporating".

### Composite fabrication

The epoxy/ nickel oxide composites with 5, 7, 9 and 11 wt% were made in three different thicknesses (2, 4 and 6 mm) with the 3cm×3cm dimension. The reported results represent an average of three different specimens for each thickness. Fabrication procedure is the same as our previous work ^17^. Exclusion criteria for fabricated composites (samples) were defined as follows: visible bubbles, deposition and agglomeration of nickel oxide nanoparticle in the samples and unequal in thickness.

### Composite characterization

According to transmission / reflection method, shielding effectiveness (SE) in the X-band frequency range (8-12.5 GHz) were measured by scattering parameters^18^ that directly are given by the set-up that including: Waveguides corresponding to the WR-90 standards are used; the electromagnetic ﬁelds are generated and recorded by an Agilent 8510C 2-port Vector Network Analyzer (Agilent Technologies, Santa Clara, USA). The setup is calibrated by Agilent 8517B S- Parameter test set (Agilent Technologies, Santa Clara, USA)., coaxial calibration carried out by 3.5 mm calibration kit to cancel out the effects of cables and connectors as well as of the waveguide transition.

SE describes the performance of the shield and is defined as: the ratio of measured power before and after the shield is placed in the electromagnetic field^10^. Since it is difficult to measure the electromagnetic properties directly, the SE can be expressed in terms of S-parameters (scattering parameters). During measurement, the transmitted and reﬂected power density is detected through scattering parameters (Sii) and recorded in relation to the incident power density Eqs. (1) and SE is obtained through the Eqs.(2)

T=|S_21_|^2^=|S_12_|^2^ Eq. (1)

SE_Total,dB_=10 Log_10_(1-T) Eq. (2)

Where:* T* is transmitted power density and S21 and S12 are the amount of transmitting energy ^11^. The input power used for all tests was 10 dBm, corresponding to 10 mw. The results were obtained in decibels, and then transformed into percentage‏ using the Eqs. (3) ^19^:

Eq.(3) $$SE(+dB)=10\log\frac{p_{i}}{p_{t}}\to SE_{\%}=\left(1-\left(\frac{1}{10^{\frac{SE}{10}}}\right)\right)\times 100$$

The type of the applied nano powders’ metallic elements was determined by X-ray diffraction (XRD, X Pert MPD‏, Philips Co. Netherlands) at 40 kV and 40 mA with Cu radiation analysis as a commonly used technique for characterization in polymer composite research. Eventually, morphological and structural information was obtained through Field Emission Scanning Electron Microscope (FESEM) S-4160 model (Hitachi Co., Japan). To characterize the distribution of embedded nanoparticles. A thin layer of gold was coated over the specimens to improve the electric conductivity needed for high-quality FESEM images.

## Results

**Figure 1:**
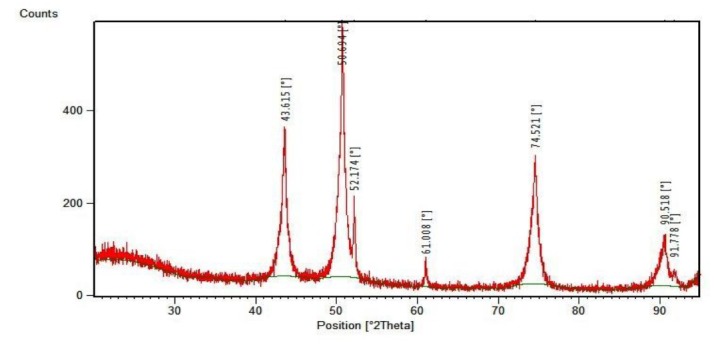
XRD spectrum of composite sample including Nickel Oxide (NiO) nanoparticles

The XRD pattern of nickel oxide was indicated in Figure 1. The nickel oxide nanoparticles had four main diffraction peaks for the Face-Centered Cubic (FCC) at 43.615^o^, 50.694^o^, 74.521^o^ and 90.518^o^, which was in accordance with the standard spectrum (JCPDS, No. 47-1049). Moreover, the XRD pattern of the nickel was detected as an impurity distinct diffraction peak. The FCC nickel at 52.174^o^, 61.008^o^ and 91.778^o^, which was in accordance with the standard spectrum (JCPDS, No. 04-0850). In addition, figure1 showed clearly that nickel, as an impurity was very low in comparison to the nickel oxide.

**Figure 2:**
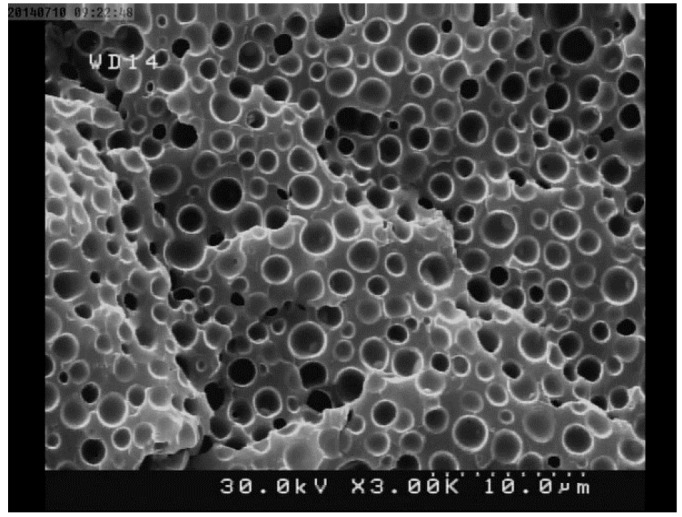
FESEM images of Epoxy resin / Nickel oxide composite, 0.5 wt. % of NiO inclusions

An FESEM image of nickel oxide nanoparticles is shown in Figure 2. This figure shows the morphology of nickel oxide particles inside the porous epoxy matrix. The particles are mostly near spherical shape. The image shows the good distribution of particles inside the epoxy matrix. However, some particles are observed as agglomerated particles, but not resulted in the loss of desirable dispersion in nanoparticles.

Table 1 shows the maximum, minimum and the average value of total shielding effectiveness for the fabricated composites in the X-band frequency ranges. This table indicates that 7%-4mmand 5%-2 mm composites achieved maximum and minimum average values of shielding effectiveness that were 66.72% and 36.24%, respectively. Also, the maximum (84.18%) and minimum (16.73%) SE values in X-band frequency range were occurred at 8.01 and 9.88 GHz that achieved by 11%-6mm and 5%-2mm composites, respectively. As the Table 1 indicates, maximum SE values were obtained by 11%-6 mm, 5%-6 mm and 11%-4 mm-fabricated composites at 8.01, 8.51 and 8.53 GHz by shielding effectiveness of 84.14%, 83.57 and 81.30%, respectively.

**Table 1:** Shielding effectiveness of epoxy resin /nickel oxide composites as functions of NiO content and shielding thicknesses (The composites were named by two characters that were thickness and filler loading (wt%).For example the composite with 4mm in thickness and 7wt% in nickel oxide loading content, was named 7%-4mm)

**Thickness (mm)**	**NiO Content (wt %)**	**SE Average** **(dB)**	**Standard deviation** **(±dB)**	**SE Average (%)**	**SE% Min** **(Frequency (GHz))**	**SE% Max** **(Frequency (GHz))**
2	5	1.95	0.56	36.24	16.73 (9.88)	51.42 (8.55)
	7	2.67	0.63	45.97	27.47 (9.86)	59.60 (8.55)
	9	2.29	0.57	40.96	23.27 (9.86)	62.63 (11.94)
	11	2.74	0.63	46.80	29.74 (9.84)	60.39 (8.55)
4	5	4.45	0.91	64.12	50.20 (11.67)	78.73 (8.53)
	7	4.78	0.92	66.72	52.95 (11.01)	80.48 (8.53)
	9	3.92	0.78	59.44	44.63 (11.01)	74.00 (8.53)
	11	4.61	1.05	65.38	49.63 (12.14)	81.30 (8.53)
6	5	4.46	1.40	64.18	42.72 (12.16)	83.57 (8.51)
	7	4.50	1.21	64.52	46.90 (12.14)	82.32 (8.51)
	9	4.49	1.27	64.43	45.60 (12.14)	82.39 (8.51)
	11	3.97	1.62	59.93	32.11 (12.12)	84.18 (8.01)

The best average of shielding effectiveness in each thickness of fabricated composites obtained by 11%-2 mm, 7%-4 mm and 7%-6 mm composites with SE values of 46.80%, 66.72% and 64.52%, respectively (Table 1).

In addition, the results in this table showed that with increasing the thickness from 2 to 4 mm (in all filler loading weights), the SE values increased noticeably. But, it was not appeared from 4 mm to 6 mm.

**Figure 3:**
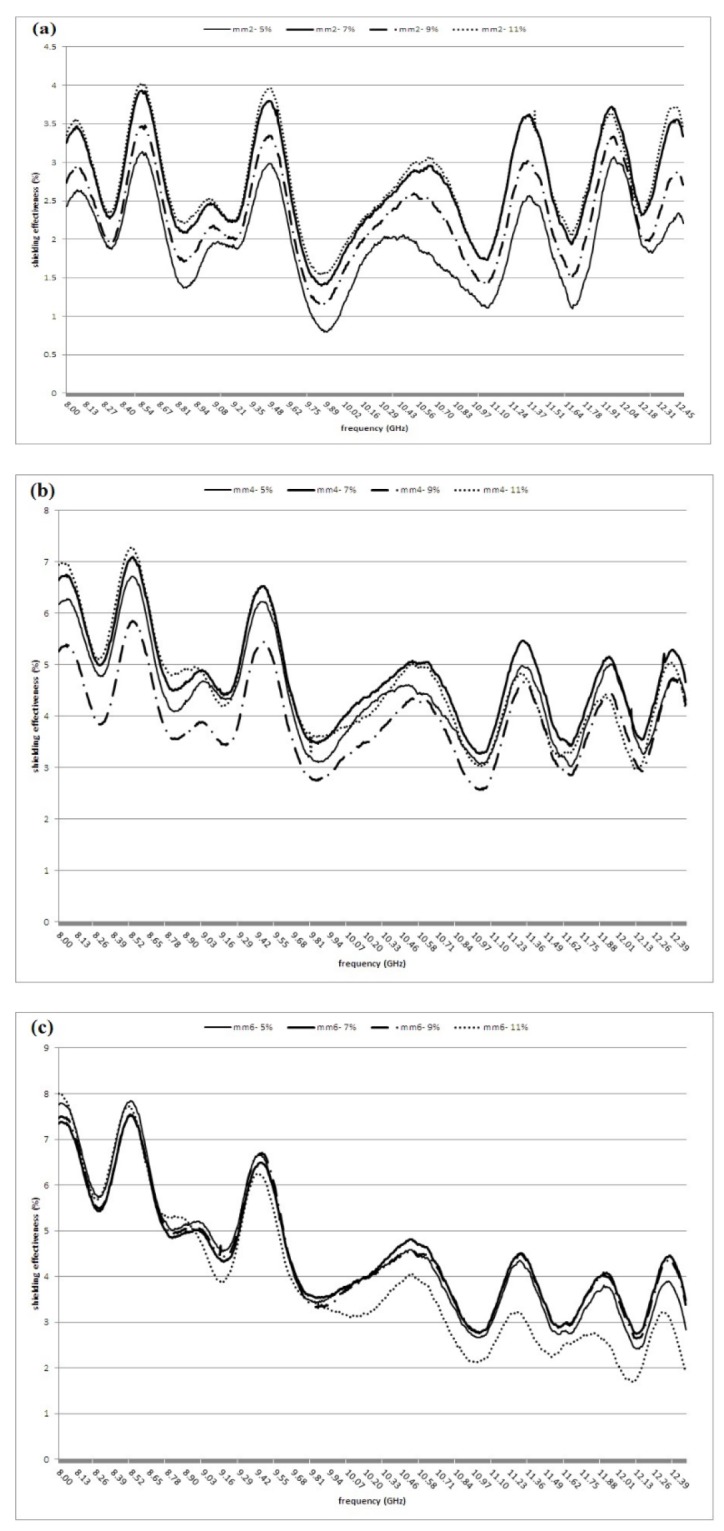
shielding effectiveness of 2 mm (a), 4 mm (b) and 6 mm (c) thickness of composites in the X-band frequency range

Figures 3 present the SE values of fabricated composites in the each frequency of X-band range. These figures graphically illustrated that there were variation in the SE values in through of the x- band frequency ranges (in all thicknesses). These variations were 16.73%-62.63%, 44.63%-81.3% and 32.11%-84.18% in 2, 4 and 6 mm thickness of composites, respectively. The variations in the SE values for the 4 mm thickness of composites were lower than other thickness (36.67% vs. 45.9% and 52.07%) (Table 1).

Figure 3 indicates that the SE values of 7%-4 mm were higher than other composites from 9.54 to 12.5 frequencies range. Also, these figures show that the SE values decreased with increasing the frequencies, only in the 6 mm thickness of composite shields.

## Discussion

In the most countries the electromagnetic radiations engross the governmental concern through their adverse health effects ^18^. The hierarchy controls that should be considered and implemented for RF exposure are elimination, substitution, engineering controls, administrative controls, and personal protective equipment ^21^. Engineering measures are the most effectiveness control to reduce or eliminate the RF / microwave exposure^1,22^.

The ICNIRP like to numerous international, governmental and regulatory sources emphasis on properly shielding as one of the engineering control methods on the Sources of RF / microwave radiation to decrease scattered radiation ^23^.

There is insufficient research to show utilization of preventive measures about microwave exposure^19^. So, with the purpose of reducing potential risks of occupational exposure, we prepared the new polymeric composite (epoxy resin/ nickel oxide nano particles) for microwave shielding in X-band frequency range that can be applied to protect workers in the workplace.

In this study, FESEM prepared the qualitative data about the degree of NiO particles dispersion in the fabricated composites^24^. The structural and morphological characterization showed the good distribution of NiO particles inside the porous epoxy. The image of the specimens containing nickel oxide showed that separable particles sizes were near the NiO nanoparticles size (15-35 nm) so, the desirable dispersion of NiO nanoparticles were observed (Figure 2). The XRD spectrums showed the existence of the nickel oxide. In addition, The XRD pattern detected slight content of nickel as an impurity distinct diffraction peak.

In addition, the results showed that with increasing the thickness from 2 to 4 mm composites thickness, the SE values increased noticeably. However, it was not appeared from 4 mm to 6 mm. Therefore, an increase in thickness was useful up to 4 mm in the present work (Table 1).

Prior research reported the negligible or limited effect of thickness on SE. They indicated that the thickness in 1, 2 and 12, 13, 14 mm showed the negligible and limited effect on the reﬂection loss in nickel ferrite nano-composite^25^. Also, a study investigated on microwave absorption of epoxy-silicone ﬁlled with multi-walled carbon nanotubes and carbonyl iron particles in 2-18 GHz frequency range. They indicated that with increasing the thickness, the SE values be increased in 2-12 GHz frequency range. But, the increasing effect of thickness was not observed in 12-18 GHz frequency range^26^.

The finding from SE measurement of fabricated composites indicated that the average of SE in 2 mm composites thickness were less than 50% and in 4 composites thicknesses were more than 50% (Table 1). However, the average of SE value in 6mm composites did not increase noticeably in comparison to 4 mm composites.

In the health physics, the thickness of a shield that reduces the radiation level to 50% of the initial level called to the half value layer (H.V.L). As yet, this attenuator layers have defined for ionizing radiations, especially X-ray radiation ^27^. Our study presents this description for X-band frequency range as non-ionizing radiation. Therefore, according to our results, the 4 and 6 mm thicknesses of fabricated composite shield can be applied as attenuator layer due to their attenuation values that were more than half value layer. These composites (in all filler loading) reduced the studied radiation to less than 50% of its incident energy.

The results indicated that the best average of SE value (66.72%) was occurred about the 7%-4 mm composite and the range of SE for this composite were 52.95% to 80.84%, respectively (Table 1). This value was more than some prior studies. A previous study was conducted on a flexible microwave absorber based on nickel ferrite nano-composite that obtained 10.88%-55.33% SE value range^28^. Another research investigated the microwave absorption of nanoparticle composites based on M‏ type ferrite with 10.87%-36.9% SE value range^29^. Also, a prior study report 10%-75% SE value range for nano-composites with nanosized-Fe3O4 and Fe as the fillers and epoxy as the matrix^30^.

Our result demonstrated that the 7%-4 mm composite on average attenuated the incident microwave energy more than a H.V.L. In other words, this attenuation level is from H.V.L to more. Therefore, the 7%-4 mm composite is the best shield for the X-band frequency range in present study.

Electromagnetic shielding properties of the ﬁller loaded polymer composites depend on various factors such as; polymer matrix, process conditions, filler type, conductivity and loading level of ﬁller^27^.

In recent years, high content of different metal based nanoparticles such as ferrites, metals, metal oxides and metal alloys have been extensively used as filler ^11^ in combination together or other carbon nanoparticle such as carbon nanotube, graphite, graphene , nano carbon fiber, carbon black^31^. Therefore, the SE (66.72%) of our preferable shielding composite (7%-4 mm) was noticeable, with attention to low content (7 wt %) of a metal based filler.

This study illustrated the variations in the SE values in through of the X- band frequency ranges. Morphology and electrical properties of composites are the effective factors on SE and influenced by nanoparticle dispersion and distribution in composite material ^24^.Some previous studies showed that the increasing mixing time caused the mixing energy and better dispersion‏ ^24^that lead to increase in values and decrease in variation of SE in X-band frequency range ^32^ . Therefore, the variations of SE in our study depend on the mixing time as the one of the important processing methods and processing conditions.

In addition, some of fabricated composites in our study were able to attenuate extremely the incident microwave energy at the three frequencies that including 8.01, 8.51 and 8.53 GHz by that were 11%-6 mm, 5%-6 mm and 11%-4 mm fabricated composites, respectively. The SE for mentioned composites was 84.14%, 83.57 and 81.30%, respectively. So, these composites could be applied as a specific shield for mentioned frequencies to reduce the radiation at least to 20% of its incident energy.

We cannot found similar studies on provision or designing the microwave shield (especially polymeric composite) to attenuate the X-band frequency range with the radiation protection approach for workplace. It is one of the limitations in our study. Few studies were found that investigate the shielding material or RF protection practices for the workplace conducted on lower frequency ranges (lower than GHz) ,,^33^.

Wright et al. studied on the control of radiation in 30 workplaces. They found that 72% of operators and 35% of bystanders, the spatially averaged /exposure exceeded the exposure limits. Task rotation as an administrative control was used to limit exposure of operators .they reported that there was lack of knowledge about RF shielding practices in industry^33^.

Kopple et al. tested various mitigation materials such as iron bar, Iron wire netting, Graphite paint and Metallic frame in the RF range spectrum (800-2500MHz) for the workplaces. They showed the best results (14.4 dB) to be attained by the 2 layers metalized canvas (curtain) ^5^ equal to 96.37% attenuation in incident radiation according to SE% equation ^16,31^. However, metalized canvas is the most expensive ^5^ and it is an important disadvantage for radiation shielding in the workplace. In addition, they indicated that the shielding effectiveness for graphite paint and metallic frame were 0.5 and 0.3 dB respectively ^5^ equal to 10.87% and 7.15%, respectively according to SE% equation ^15,31^. Therefore, attenuation value for these materials is inconsiderable.

In their study, the SE for Iron bars10 cm gap- grounded, Iron bars 20 cm gap- grounded and Iron bar with one in front of the meter- grounded were 5.9 ,1.5 and 1.8 dB, respectively^5^ that is equal to 74.3% , 43.76 and 33.93%, respectively^16,31^. Our results indicated that the SE for 7%-4 mm composite (66.72%) is near to Iron bars (10cm gap, grounded).

Shields made of metal are heavy ^9^whereas lightweight is one of the advantages for the composite shields, in our study. For instance, the 7%-4 mm composite with 30×30×4mm dimension was 5.31 gr in weight, but this weight for the same size of iron plate as solid metal sheets is 31.5 gr according to its density that is 5.93 times heavier than our composite.

With attention to appropriate SE of 7%-4 mm composite and other its preferable properties such as low cost and weight, excellent mechanical properties, chemical and heat stability , wet and chemical resistance antibacterial properties, wet and chemical resistance processing ability, resistance to corrosion^13,34^ due to present the epoxy in its structure, it can be introduced as suitable alternative microwave shield in radiation protection topics, to reduce occupational exposure in the workplace.

Our fabricated shield can be applied as architectural shield in the workplace. Therefore, it would be built into the walls of the procedure room and can be useful to isolate and insulate workers from microwave sources. It is recommended that efficacy of our fabricated composites be developed and investigate in the fields by future studies.

## Conclusions

In this study, a new microwave shield (using Epoxy resin / Nano nickel oxide) prepare to reduce exposure of X-band frequency range with occupational safety approach. The results indicated that the 7%-4 mm composite can be introduced as a suitable alternative microwave shield in radiation protection topics in order to its proper SE and other preferable properties such as low cost and weight, resistance to corrosion and etc. In addition, the 11%-6 mm, 5%-6 mm and 11%-4 mm-fabricated composites were able to attenuate extremely the incident microwave energy at 8.01, 8.51 and 8.53 GHz by SE of 84.14%, 83.57 and 81.30%, respectively. Therefore, these composites could be applied as a specific shield for mentioned frequencies to decline the radiation at least to 20% of its incident energy. It is necessary to develop and investigate the efficacy of the fabricated composites in the workplace fields by future studies.

## Acknowledgments

The authors would like to thank Tarbiat Modares University for their financial support and Mohammad Hassan Moeini for technical support. The insightful comments of the reviewers are greatly acknowledged

## Conflict of interest statement

The authors declare that they have no competing interests.

## Highlights

- Epoxy resin/Nano nickel oxide composite could be ap-plied as a suitable alternative microwave shield.
- Increasing the thickness, had limited efficacy on shielding effectiveness.
- Determined fabricated composites could be applied as a specific shield for some frequencies.
